# Neural regulation of the thymus: past, current, and future perspectives

**DOI:** 10.3389/fimmu.2025.1552979

**Published:** 2025-02-19

**Authors:** Randall S. Carpenter, Maria K. Lagou, George S. Karagiannis, Maria Maryanovich

**Affiliations:** ^1^ Department of Cell Biology, Albert Einstein College of Medicine, Bronx, NY, United States; ^2^ Ruth L. and David S. Gottesman Institute for Stem Cell and Regenerative Medicine, Albert Einstein College of Medicine, Bronx, NY, United States; ^3^ Department of Microbiology and Immunology, Albert Einstein College of Medicine, Bronx, NY, United States; ^4^ Tumor Microenvironment Program, Montefiore-Einstein Comprehensive Cancer Center, Bronx, NY, United States; ^5^ Gruss-Lipper Biophotonics Center, Albert Einstein College of Medicine, Bronx, NY, United States; ^6^ Cancer Dormancy Institute, Montefiore-Einstein Comprehensive Cancer Center, Bronx, NY, United States; ^7^ Integrated Imaging Program for Cancer Research, Albert Einstein College of Medicine, Bronx, NY, United States; ^8^ The Marilyn and Stanely M. Katz Institute for Immunotherapy for Cancer and Inflammatory Disorders, Montefiore-Einstein Comprehensive Cancer Center, Bronx, NY, United States

**Keywords:** sympathetic nervous system (SNS), thymus aging, beta-adrenergic signaling pathway, innervation of the thymus, age-related thymic involution, thymus involution, T cell development, thymopoiesis

## Abstract

The thymus is a primary lymphoid organ critical for the development of mature T cells from hematopoietic progenitors. A highly structured organ, the thymus contains distinct regions, precise cytoarchitecture, and molecular signals tightly regulating thymopoiesis. Although the above are well-understood, the structural and functional implications of thymic innervation are largely neglected. In general, neural regulation has become increasingly identified as a critical component of immune cell development and function. The central nervous system (CNS) in the brain coordinates these immunological responses both by direct innervation through peripheral nerves and by neuroendocrine signaling. Yet how these signals, particularly direct neural innervation, may regulate the thymus biology is unclear and understudied. In this review, we highlight historical and current data demonstrating direct neural input to the thymus and assess current evidence of the neural regulation of thymopoiesis. We further discuss the current knowledge gaps and summarize recent advances in techniques that could be used to study how nerves regulate the thymic microenvironment.

## Introduction

The thymus is an encapsulated primary lymphoid organ responsible for T cell development. It is strictly compartmentalized into an outer cortex and an inner medulla, organized into distinct functional units known as thymic lobules. Early thymocyte (T cell) development starts in the cortex of the lobule, the site of thymocyte progenitor homing, T cell lineage commitment, T cell receptor (TCR) chain gene rearrangement, and positive selection (1, 2). These positively selected thymocytes are then chemoattracted and migrate into the medulla, whose primary role is to eliminate self-reactive thymocytes through promiscuous gene expression of endogenous self-antigens and the process of negative selection. Eventually, functionally mature yet still naïve CD4^+^ or CD8^+^ T cells emigrate to the peripheral blood and home to secondary lymphoid organs (1).

Early studies found that the education of thymocytes is facilitated by its stroma, primarily via the thymic epithelial cells (TECs) (1). To date, the TEC population has been most extensively studied among thymic niche cells. Single-cell transcriptomic studies have allowed in-depth characterization and identification of heterogeneous cortical and medullary TEC (cTEC and mTEC, respectively) populations in the thymus, highlighting their complexity (1, 3). Elegant *in vivo* cell fate-mapping studies have identified at least two bipotent TEC progenitors in the adult thymus exerting regenerative capacity, with the first mainly presenting with a cTEC bias during early life, while the second being mTEC-biased and preserved until later in life (3). While the heterogeneity of cortical TECs (cTECs) is still underappreciated, recent evidence has indicated a progressively linear differentiation pathway starting from the bipotent cTEC-biased progenitor and leading to well-differentiated cTEC expressing an abundance of homing chemokines and signals essential for T cell development (1). Mature cTEC can further become terminally differentiated thymic nurse cells (TNCs) that form specialized large three-dimensional cellular microenvironments, encapsulating and nurturing immature thymocytes with higher efficiency during positive selection (1). However, studies have shown that mTECs are highly heterogeneous, dividing into different subsets. Although the mTEC developmental pathway is rather complex, the accepted paradigm identifies an early divergence of bipotent mTEC-biased progenitors into the committed mTEC^lo^ and mTEC^hi^ subsets, with the former expressing homing chemokines for the positively selected thymocytes, while the latter presenting self-antigens to thymocytes in an autoimmune regulator (AIRE)-dependent manner for the establishment of self-tolerance (1). Remarkably, in-depth molecular characterization of mTEC subsets has also revealed a subset of “terminally differentiated” post-AIRE population, termed “mimetic cells”, which undergo developmental trajectories of and mirror various specialized peripheral cells to highly express narrower repertoires of highly specific tissue-restricted antigens (4).

Besides TECs, the thymic niche also includes other immune cell types such as macrophages, innate lymphoid cells (ILCs), natural killer T (NKT) cells, dendritic cells, and B cells, which play essential roles in positive selection and tolerance induction (5, 6). Moreover, TEC maintenance is supported by thymic stromal cells of mesenchymal origin, such as fibroblasts. More recently, fibroblasts have been described to have diverse and critical roles in the thymic microenvironment (7–9). Divided into capsular and medullary subsets based on their localization, fibroblasts play crucial roles during thymic development, TEC maintenance, and tolerance induction. Recent transcriptomic studies have revealed that by inducing type 2 immune response, fibroblasts can assist in thymic regeneration, showcasing a deeper and more complex positional role in the thymic niche (10).

Despite the significant strides made in understanding the thymic microenvironment and the regulatory networks within, one critical aspect remains relatively unexplored: the neural regulation of the thymus. In many organs and organ systems, peripheral nerves orchestrate both critical and finetuning processes, typically by regulating the structure and function of stromal components. Although the presence of nerves in the thymus has been documented since the early 20^th^ century, their relationship with the aforementioned stromal components and their functional implications in thymopoiesis remain largely enigmatic. In this review, we examine historical and contemporary evidence of thymic innervation and delve into its potential implications for T cell development and self-tolerance. Additionally, we will highlight the current gaps in knowledge and explore emerging technologies poised to unravel how thymic neural networks and their downstream mechanisms contribute to thymopoiesis.

## Evidence of direct neural innervation of the thymus

In 1929, Pine & Majman reported on the direct innervation of the thymus, characterizing nerve bundles running along large vessels and forming plexuses that envelop the vascular wall (11). They characterized these nerves as sympathetic in origin due to their lack of myelination and size. Nearly fifty years later, in the 1980s, Williams & Felten (12) described “varicose fibers” that associated with arteries and arterioles, with “plexuses which lay in the adventitia outside of the external limiting membrane.” Sparse innervation of the parenchyma was also noted without any vascular association. Soon after, Bulloch & Pomerantz observed acetylcholinesterase (AChE)-positive fibers on the surface of the thymus, while catecholamine-positive fibers from the sympathetic nervous system (SNS) were noted along large blood vessels, the capsule, and intralobular septa of the thymus (13). Further noted were the many nerve fibers associated with the vasculature of the cortico-medullary junction (CMJ), with vasculature-free fibers in the parenchyma of both the medulla and cortex. Felten & colleagues followed up with an in-depth assessment of the SNS innervation to the thymus (14). Their studies demonstrated that sympathetic nerve fibers run along the capsule, entering the thymic parenchyma into the cortex and along vessels that dive into the thymus directly from the capsule. They then hypothesized that these nerves release neurotransmitters into the thymus to regulate blood flow, directly activate adrenergic receptors on lymphocytes, or indirectly influence thymocytes by activating adrenergic receptors on supporting cells.

**Figure 1:**
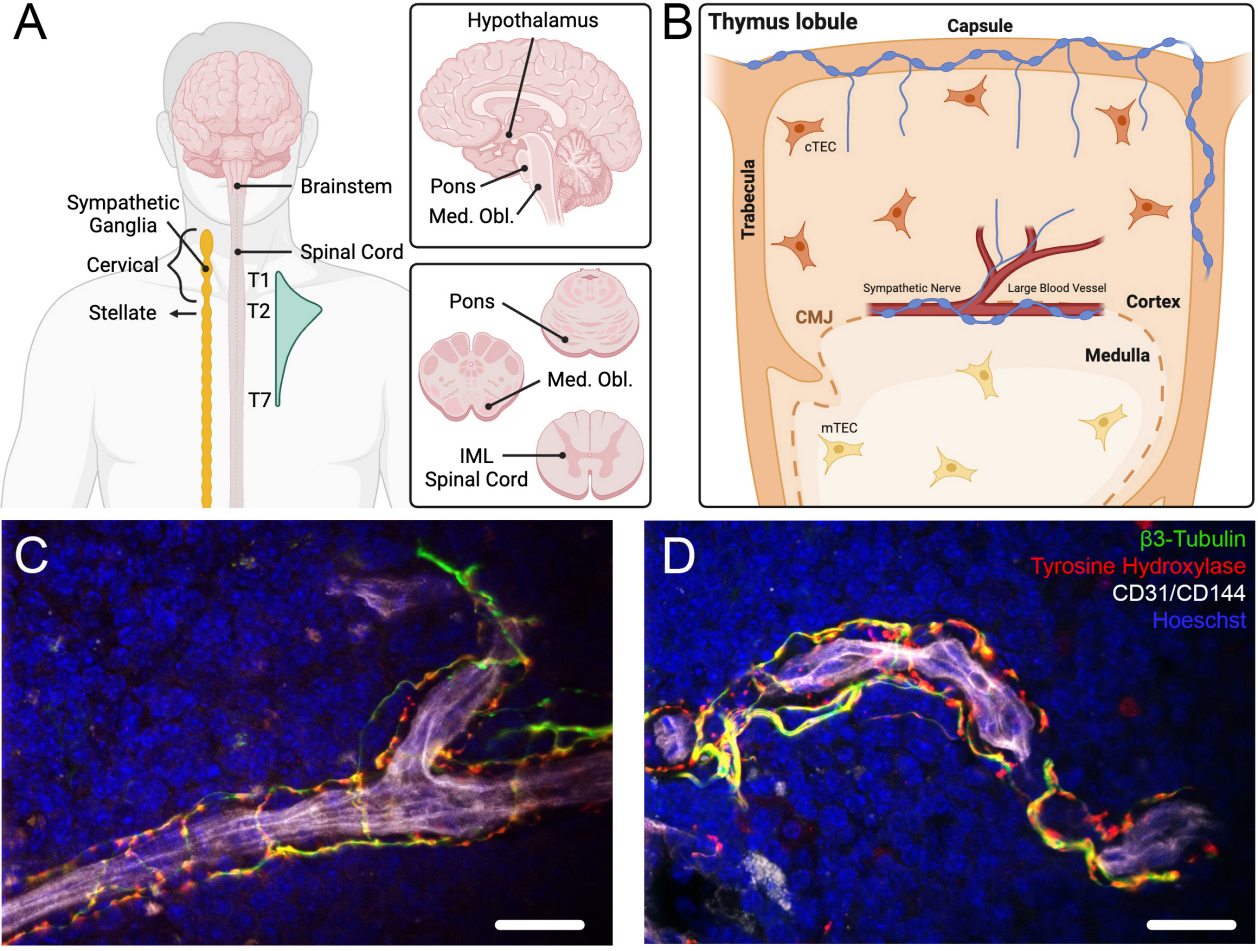
Innervation of the thymus. **(A)** Diagrams of the brain, brain stem, spinal cord, and sympathetic ganglia summarizing the origin of neural innervation of the thymus. The thymus is innervated by sympathetic nerves that arise from the cervical and stellate ganglia of the sympathetic trunk, which are connected to cells in the intermediolateral cell column (IML) of the thoracic spinal cord from T1 to T7 (peak connectivity at the T2 spinal level). Other brain regions connected functionally to thymic sympathetic neurons include the pons, medulla oblongata (med. obl.), and hypothalamus. **(B)** Diagram of a thymic lobule, demonstrating sympathetic innervation along large vessels that reside in the cortico-medullary junction (CMJ), with some fibers branching into the cortical parenchyma. Sympathetic nerves also innervate the capsule, projecting individual nerve fibers into the subcapsular cortex, and along the trabeculae. **(C, D)** Confocal images showing sympathetic innervation (β3-Tubulin^+^/TH^+^ nerves) of blood vessels (CD31^+^/CD144^+^) in a young adult mouse thymus. Scale bar = 25μm. Panels **(A, B)** created with BioRender.com.

These early studies demonstrated the presence of SNS fibers in the thymus, although their origin was not resolved. At first, retrograde tracing labeled neurons in the spinal cord and brainstem (15). However, a later study refuted this, demonstrating that SNS nerves were derived from the sympathetic chain, including the superior cervical ganglia (16). Indeed, retrograde labeling observed in the spinal cord or brainstem was caused by contaminating nearby muscle tissue and other structures with retrograde tracer (16). However, another tracing study by Tollefson & Bulloch using fluorogold again demonstrated labeling in the cervical spinal cord and brainstem, concluding actual labeling of efferent projections of the vagus nerve (17). Other studies have not detected gross anatomical connections between the vagus/phrenic nerves and the thymus (16), although connections between the thymus and the stellate ganglia of the sympathetic chain were also reported (13). Thus, the relative contribution of the cervical and stellate ganglia to thymic SNS innervation is currently unclear, although they both partially contribute (**Figure 1A**). Importantly, the study by Tollefson & Bulloch did not address two issues brought up by Nance and colleagues: 1. the lack of gross anatomical connections between the thymus and vagus/phrenic nerves, and 2. the unaltered AChE labeling within the thymus following unilateral cervical vagotomy (16). These observations imply an absence of direct parasympathetic input via the vagus nerve. Tollefson & Bulloch did cede that small amounts of tracer could traverse lymphatics or leak to other tissues, possibly causing the labeling in the brain stem and cervical spinal cord, but remained adamant regarding parasympathetic input to the thymus.

Viral tracing of thymic sympathetic nerves demonstrated connectivity to the intermediolateral cell column of the spinal cord between T1 and T7 thoracic segments (**Figure 1A**), with the highest density occurring at the T2 thoracic segment (18). Longer periods of tracing labeled cells in the intercalated nucleus and central autonomic nucleus of the spinal cord and labeled brainstem neurons in the pons and medulla oblongata (**Figure 1A**). The vagus nerve nuclei were not labeled, further supporting that the thymus receives little to no input from the parasympathetic nervous system (PNS). Tracer labeling was also found in multiple hypothalamic nuclei (**Figure 1A**), including the paraventricular nucleus, a master regulator of autonomic function (19).

In summary, early anatomical studies revealed that sympathetic nerves enter the thymus along the vascular network, with singular nerves occasionally extending from vessel branches into the adjacent cortical parenchyma (**Figure 1B**). Indeed, many innervated vessels strictly localize to the CMJ. Conversely, capsular nerves project from the capsule into the subcapsular cortex, some by following along the septa and vessels, while others by circumventing the vasculature and penetrating directly into the cortical parenchyma. Kendall and Al-Shawaf have also shown that the subcortical region is densely innervated by finely varicose fibers that often do not associate with capillaries (20). While some studies found fibers potentially related to parasympathetic innervation (13, 17, 20), many others suggest that parasympathetic innervation is non-existent, attributing the positive results to unintended technical errors (12, 14, 16, 18).

Interestingly, some nerves, established as SNS nerves from these prior studies, also express peptidergic neurotransmitters neuropeptide Y (NPY), vasoactive intestinal peptide (VIP), and calcitonin gene-related peptide (CGRP) (12, 14, 20–22). While sympathetic nerves can produce NPY, VIP is usually associated with the brain and digestive system, and as such, the neural source of VIP in the thymus is currently unclear. VIP and NPY both regulate gut immunity and may similarly act on developing T cells in the thymus (23, 24). Finally, CGRP is typically associated with nociceptive nerves, but no direct evidence of sensory/nociceptive innervation of the thymus is available besides positive CGRP immunoreactivity (21, 22).

A recent immunohistological study using the common neuronal/axonal marker neurofilament (NF) reported remarkable labeling in the cortex and medulla of the thymus (25). While some NF signal colocalized with large CD31^+^ blood vessels, much of the labeling in both cortex and medulla formed a ramified cellular pattern similar to intermediate filament (e.g., cytokeratin) labeling of TECs. Therefore, whether these NF-expressing structures are indeed nerves or define other thymic cells expressing NF as part of their normal developmental program remains unclear. In the same study, the authors did not incorporate other general or axon-specific markers to verify the precise cellular source of NF immunolabeling. These results are in contrast with previous literature as well as with our observations demonstrating that the general axon marker β3-Tubulin and the SNS-specific marker tyrosine hydroxylase (TH), both labeled sympathetic nerves associated with large blood vessels in murine thymic tissue (**Figures 1C, D**).

## Non-neural sources of neurotransmitter production and signaling

Given that some neurotransmitters can be generated by non-neuronal cells, including lymphocytes, to modulate immune cell functions (26–28), it is important to consider neurotransmitter production and signaling within the thymic environment in the absence of parental nerves. This possibility may justify why certain neuronal markers are identified in the thymus without clear input from nerve structures. For example, acetylcholine may be locally synthesized within the thymus by thymocytes and other cell types (29, 30), which could further explain the positive labeling of AChE and choline acetyltransferase (ChAT) without documented existence of the corresponding nerves. Indeed, using ChAT-GFP mice, a subset of TECs expressing the epithelial marker cytokeratin were also identified to be cholinergic (31). These cells were closely associated with nicotinic acetylcholine receptor-expressing cells and denuded from direct neural innervation, indicating a paracrine signaling mechanism. However, immunolabeling for neural markers within TECs could be due to the presence of thymic mimetic cells, a subset of TECs that express tissue-restricted antigens (32). Indeed, a previous report found a small but significant population of TECs expressing neural markers (33).

Interestingly, the murine thymus expresses high levels of phenylethanolamine N-methyltransferase (PNMT), the key enzyme involved in the conversion of norepinephrine (NE) to epinephrine (34). PNMT levels in the cortex were doubled than those in the medulla, and overall PNMT levels were equivalent to those seen in the brainstem of the CNS. Thus, it is intriguing to speculate that cells in the thymus can uptake NE released from sympathetic nerves, convert it to epinephrine autonomously, and subsequently utilize epinephrine as a secondary signaling cascade throughout the thymic parenchyma.

Isolated thymocytes stimulated with concanavalin A or IL-2 show a concentration and time-dependent increase in intracellular and extracellular CGRP (35). Stimulated thymocytes increase CGRP mRNA expression. Notably, exogenous CGRP increased proliferation and IL-2 production during concanavalin A stimulation, demonstrating that CGRP can enhance thymocyte responses. Serotonergic and dopaminergic signaling components have also been found in the thymus, with potential roles for their signaling in mediating T cell development (36–38). Thymocytes and thymic niche cells can also express metabotropic glutamate receptors, exhibiting differential levels of surface expression depending on the stage of thymocyte development (39). Thymocytes also express α- and β-adrenergic receptors (40, 41). Together, these studies indicate that neurotransmitters are likely co-opted by cells in the thymus to coordinate T cell development even in the absence of the corresponding parental nerves. However, whether paracrine neurotransmitter production or release in the thymus is influenced by brain activity, either through direct neural or indirect neuroendocrine signals, is largely unknown.

## A functional role for neural signals in regulating thymopoiesis

What is the role of extensive neural innervation in the thymus? While many anatomical studies speculate on potential functions, few experimental studies exist. Thymic sympathetic nerves were shown to release NE in response to field stimulation in rats (42). This stimulation was ablated in calcium-free conditions and in the presence of the sodium channel blocker tetrodotoxin, indicating that NE release was axonal in origin. Patch-clamp recording of thymocytes demonstrated that NE inhibits voltage-gated potassium channels in a dose-dependent manner (42). Thus, NE is likely released through sympathetic nerve terminals in response to increased neuronal activity, where it acts locally through diffusion to nearby cell types, including thymocytes and other stromal cells, such as TECs. Interestingly, the muscarinic acetylcholine receptor agonist oxotremorine and prostaglandin E2 were both able to inhibit stimulation-dependent NE release in rat thymus sections (43), suggesting that other signals within the thymus may modulate sympathetic activity. Another critical discovery from these studies is that NE released by sympathetic nerves within the thymus binds to α2c-adrenergic receptors directly on the nerves, generating a tonic inhibitory feedback loop that may help mediate sympathetic tone.

Although stress-mediated thymic atrophy is typically associated with glucocorticoid-mediated apoptosis of DP thymocytes (44), there is critical evidence that activation of SNS nerves may also independently contribute to this phenotype via NE release and its direct impact on thymocytes. Isoprenaline, a β1 and β2 adrenergic receptor-specific agonist, induced apoptosis in double-positive (DP) thymocytes freshly isolated from adult thymus through the activation of Src tyrosine kinases (45). Thymocyte gene expression may also be altered by catecholamines, as Thy-1 expression on thymocytes is reduced by NE signaling through β-adrenergic receptor signaling, suggesting mRNA destabilization is rapidly influenced by these signals (46, 47). Catecholamines may also inhibit thymocyte activation by concanavalin A, although the reported mechanism seems independent of classical receptor activation, as catecholamine-mediated inhibition was not blocked using adrenergic receptor antagonists (48). In addition, neuropeptide Y was reported to alter chemotaxis and adherence of thymocytes in *in vitro* conditions (49). As discussed above, most studies examine a direct effect of NE and other neurotransmitter signaling on thymocytes and neglect potential interactions within the thymic microenvironment involving epithelial and non-epithelial stromal cells. In one prominent study, isolated rat TECs responded to NE by increasing IL-6, suggesting the ability to alter their cytokine production in response to adrenergic signaling (50). In another study, *in vivo* and *in vitro* treatment with the muscarinic acetylcholine antagonist atropine promoted apoptosis of thymocytes, which only occurred in the presence of TECs (29). Thymocytes may directly produce acetylcholine as they express high levels of ChAT mRNA, a key enzyme that produces acetylcholine (29). Thus, thymocytes may use acetylcholine to signal TECs to promote their survival. A major limitation to many of the discussed experiments is the use of isolated primary thymocytes, extracted from the thymic microenvironment and relevant signals that regulate thymopoiesis and treated *in vitro* with supraphysiological levels of neurotransmitters and/or respective agonists/antagonists. To understand their physiological roles, neurotransmitter signaling should be studied in the *in vivo* complex structure of the thymic microenvironment. While little is known, a few studies point to their possible importance in thymus function and T cell development.

A study using a mouse model of Krabbe disease, a progressive demyelinating disease resulting in autonomic denervation of lymphoid organs, demonstrated that while lymphoid organs were normal at postnatal day 15, rapid thymic involution occurred, primarily due to thymocyte apoptosis (51). This was associated with the loss of sympathetic nerve fibers and altered β-adrenergic receptor expression, suggesting that thymic SNS innervation may be critical in regulating thymopoiesis. The depletion of neural crest-derived cells, which include sympathetic nerves in addition to a subset of mesenchymal cells (expressing platelet-derived growth factor receptors α and β, and α-smooth muscle actin) typically involving fibroblasts and pericytes, also results in rapid thymic involution (52). However, this model used a *Wnt1*-Cre deletion line, which recombines in non-neural cells throughout the body and, as such, is not specific to the thymus. Along the same lines, using 6-hydroxydopamine (6-OHDA), known to induce global peripheral sympathectomy, also resulted in significant loss of thymocytes. However, this was likely not due to the specific loss of β-adrenergic signaling, as two week-long daily injections of propranolol and cyanopindolol did not replicate this phenotype. Indeed, 6-OHDA primarily impacted the cortex by inducing apoptosis in DP thymocytes (20, 53, 54). These studies suggest a potential beneficial role for catecholamine signaling in the thymus that, when lost through disease or genetic deletion, may impair thymus function.

How neural innervation and activity are altered in physiological aging or a diseased context is vastly understudied. Studies utilizing cuprizone treatment and experimental autoimmune encephalitis models of multiple sclerosis have shown that these models are associated with thymic atrophy and thymocyte apoptosis (55–59). Whether this is due to altered CNS-thymus crosstalk is unclear. However, a recent publication examined thymic innervation in response to experimental autoimmune encephalitis (60). The authors used multiple markers of neurons and axons, with data reporting increased nerve fiber density in diseased mice. In this study, however, immunostaining often does not appear to be axonal, and retrograde tracing was performed with an unidentified tracer, raising concerns about data interpretation. Nevertheless, the study reported reduced tracer uptake in diseased mice, further implying changes in the intrathymic innervation networks or the retrograde transport capacity, although the possibility of technical artifacts cannot be excluded.

Aging is associated with altered neural innervation of several hematopoietic and lymphoid organs. Alterations in bone marrow innervation was reported to be associated with hematopoietic aging (61–63). In particular, we and others have shown that loss of bone marrow innervation drives aging-like phenotypes in HSCs (61). How aging alters thymic innervation and function is less known, but several studies of nerve fiber density and catecholamine measurements exist. For instance, nerve fiber density is increased in the aged thymus (64–66). This increase in density is likely a result of thymic involution due to progressive loss of cellularity. Yet, one study found a potential increase in total innervation with unchanged sympathetic innervation (67), raising the possibility that specific nerve subsets may have different responses to aging. Catecholamine measurements in the aged rat and mouse thymus are either maintained (65) or elevated (64, 66, 67), suggesting that nerves continue functioning to some degree in the aged thymus. However, whether any increases in NE content are due to heightened sympathetic tone, impaired NE reuptake, non-neuronal sources, or modulation by other means is currently unclear.

Changes in thymus structure during aging could contribute to altered neural signaling and thymic involution. However, only a few functional studies assess the role of NE signaling in the aged thymus. In one such study, long-term β-adrenergic blockade with nadolol increased DN and SP8 cells but decreased DP cells in aged mice (68). In another study, 14-day treatment with the non-selective beta blocker propranolol increased the numbers of SP4 and SP8 thymocytes and improved Thy-1 surface expression (41). Whether blockade of adrenergic signaling in the aged thymus leads to improved T cell development and function is uncertain, especially as neither study provides mechanistic and further phenotypic insights.

## Models and methods to study thymic neural anatomy and function

**Figure 2:**
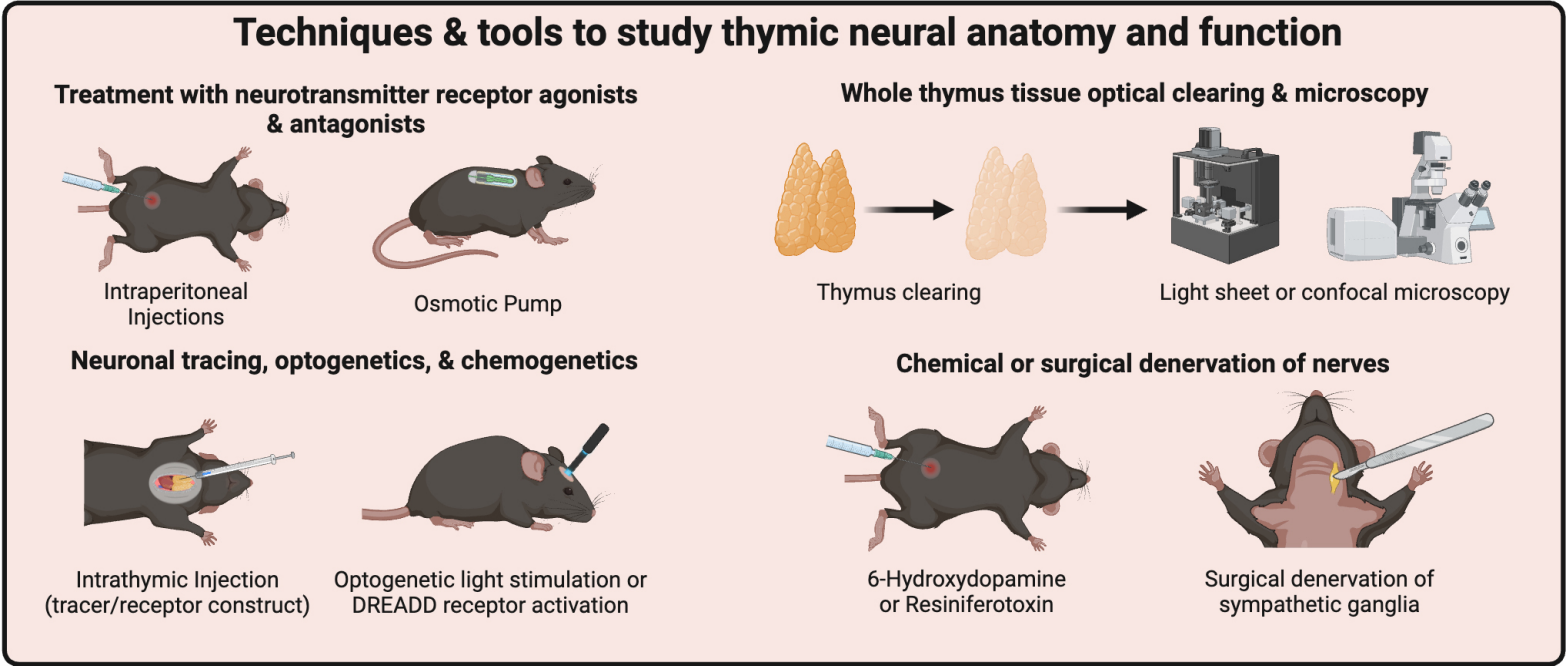
Techniques & tools to study thymic neural anatomy and function. Many neurotransmitter receptors can be targeted by treating mice with agonists or antagonists. Thymic neurocircuitry tracing and expression of optogenetic and chemogenetic receptors requires Intrathymic injections to selectively target nerves within the thymus. Next-generation optical tissue clearing and imaging techniques will provide tissue-wide mapping of neural innervation of the thymus. Finally, chemical or surgical denervation techniques may target thymic innervation systemically or locally. Created with BioRender.com.

The past couple of decades have seen the emergence of new technologies to study neural function *in vivo*, many of which can be adapted to the study of neural regulation of the thymus (**Figure 2**). However, proper implementation of these techniques and understanding their limitations is important. Here, we provide pertinent information to understand their benefits, limitations, and how these methodologies have been used to study neuro-immune interactions.

### Chemical sympathetic denervation

As previously discussed, 6-OHDA is a neurotoxin used to cause sympathectomy through oxidative stress and apoptosis of peripheral sympathetic neurons. The benefit of this technique is the ease of use, typically requiring two doses split by a couple of days (69–73). Proper handling of 6-OHDA is critical, as it oxidizes quickly, and denervation is temporary in adult mice. Adding subsequent doses every 6 days may maintain peripheral sympathetic denervation (71). Permanent denervation can also be achieved by injecting mouse pups every other day starting at postnatal day 2 for five total doses (72, 73). One weakness of this method is that denervation occurs during development, and some effects could be attributed to improper development or adaptation of tissues. As previously discussed, 6-OHDA can also have direct toxic effects on DP thymocytes (53). Thus, careful interpretation of data must be considered to understand whether the effects of 6-OHDA are indeed due to loss of innervation or other direct/indirect effects.

### Chemical sensory denervation

Resiniferatoxin (RTX) is a potent TRPV1 agonist that results in the death of peripheral sensory neurons due to calcium overload (74, 75). Mice are subcutaneously injected with three increasing doses of RTX at approximately four weeks of age to induce long-term sensory denervation (76, 77). Mice are typically used for experiments after a 3-4 week resting period. As with 6-OHDA, whether RTX can directly affect thymocytes or other TRPV1-expressing cells in the thymus is currently unknown. Further, while CGRP can be detected in the thymus, it is unclear if its source is derived from sensory nerves. In this case, RTX may be a way to deplete this specific subset of nerves, although further mechanistic insights are still required with this agent for safer data interpretation.

### Chemical agonists & antagonists

Today, many exogenous and potent agonists/antagonists are used to target neurotransmitter receptors. Some are used clinically, while others only experimentally. Some of the oldest drugs developed are β- and α-adrenergic receptors, used to control blood flow through contractility of large arteries (78, 79). Intraperitoneal injection into rodents is the standard used in pre-clinical research and may be useful in short-term experiments. However, long-term use may require long-release pellets or osmotic pumps (61) to prevent stress associated with repeated injections and uneven pharmacodynamics. Other drugs may act indirectly on specific adrenergic or cholinergic receptors, promote or prevent the release of neurotransmitters, prevent neurotransmitter reuptake by cells, or break the neurotransmitters down into inactive or partially active metabolites. A downside to using chemical agonists or antagonists *in vivo* is that they act systemically, so determining direct effects is difficult. However, these agents can be useful for *ex vivo* or *in vitro* culture systems, and many second and third-generation drugs are longer-acting and more stable than the natural neurotransmitter.

### Knockout and transgenic mice

Neural components, including neurotransmitter receptors, can be modified using genetically engineered mouse models. Germline knockout models exist for many adrenergic receptors, including α- and β-adrenergic receptors. Some receptors have floxed (gene-flanked *loxP* site) models that can be crossed with cell-specific *Cre* or *Cre-ER* (estrogen receptor) deleter lines. The benefit of these models is in conditionally identifying neurotransmitter-receptor interactions on specific thymic cells. However, they may not showcase the overall role of nerves within the thymus, which simultaneously signal to many cell types throughout the microenvironment. Other models, such as diphtheria toxin receptor mice, may allow targeted killing of specific cells (80), although this would also be systemic and not specific to the thymus. A recent publication used tyrosine hydroxylase specific *cre* (*TH-Cre*) mice crossed with a tropomyosin receptor kinase A (TrkA) mouse harboring a mutation that allows for the timed inactivation of the nerve growth factor (NGF) binding TrkA receptor, using the chemical 1NMPP1 (81). This caused a reduction in sympathetic innervation to the spleen, ultimately altering splenic immune responses. Whether this model could also result in denervation of the thymus is unclear.

### Surgical approaches

One approach to studying neural innervation of immune tissues is through direct surgical manipulation of nerve bundles or involved ganglia. Surgical denervation techniques have been described for bone marrow (61, 69), lymph nodes (82), and spleen (83). Nerves can be directly excised, while phenol/ethanol solutions can also be used to promote nerve denervation when applied directly to nerve bundles (as in the case of the spleen, where the splenic sympathetic nerve is difficult to manually excise due to its proximity of the splenic artery). The key challenge with surgical denervation of the thymus is the precise location of the afferent nerves or the associated ganglia, particularly in the mouse, and the relative inaccessibility of the thymus and stellate ganglia, which are both located inside the thoracic cavity. Further, surgical procedures require anesthesia, may induce inflammation, and may require post-surgical care that could disrupt normal physiology, requiring a recovery period before experimentation. To date, only one study suggests that, in rat, removal of the superior cervical ganglia can result in the loss of sympathetic nerves within the thymus (84).

### Optogenetic and DREADD manipulation of neural activity

Understanding structure-function relationships in neuroscience was long limited to field stimulation and broad dissection of brain regions. Optogenetics revolutionized the ability to activate or silence specific brain structures, implementing microbial opsins that can be photoactivated or silenced using light (85, 86). These tools, used to study neuron-function relationships in awake mice, have been rapidly implemented in the field. Similarly, designer receptors exclusively activated by designer drugs (DREADD receptors) are another recent advance in altering neuronal function (87, 88). These receptors bind to physiologically inert chemicals and can also be used to selectively activate or silence specific neuronal populations (87, 88). Optogenetic models are challenging to implement as they require specialized fiber optic equipment but allow finely tuned stimulation paradigms. DREADD models are relatively easy to use, only requiring intraperitoneal injections of DREADD agonists but lacking the temporal precision of optogenetics. Both models often require retroviral transfection to selectively express these receptors in their specific neurons or tissues. However, they represent significant advances in studying neural signaling within the thymus, as they can be expressed exclusively by the neurons innervating it. These techniques can be used as alternatives to, or in conjunction with, surgical/chemical denervation, transgenic mice, and systemic agonist/antagonist administration.

### Anterograde & retrograde tracers

A long-standing technique for labeling neurons and their projections, anterograde and retrograde tracers, have been used to trace the innervation of the thymus as described above. Anterograde tracing labels neuronal cell bodies to examine afferent projections (where those neurons project to), while retrograde labeling is used to identify where axons and nerve terminals arise from. Wheatgerm agglutinin (WGA) conjugated to horseradish peroxidase (HRP), Cholera toxin B subunit (CTB), and Fluorogold are common retrograde tracers, allowing easy visualization of neuronal cell bodies that project axons into the region of interest. The major limitation is that they only label neurons that take up the tracer and cannot resolve the synaptic network. To this end, certain viral tracers, including pseudorabies viruses, may be used to label synaptic networks as these viruses can “jump” from cell to cell based on their synaptic connections. A more in-depth guide to neuronal tracing is summarized elsewhere (89).

### Imaging of thymic nerves using optical tissue clearing techniques

OTC whole-tissue imaging methodologies have helped neuroscientists identify neural networks that span the entire nervous system and throughout the body (90). Such techniques incorporate tissue processing and refractive index matching to make whole tissues and, in some cases, whole animals nearly transparent for imaging (91). The number of different clearing techniques has grown exponentially, leading to improvements in methodology. However, there is no “one-size-fits-all” approach, and understanding the nuances of each technique and their potential applications can be challenging. OTC-based imaging techniques have begun to be incorporated to visualize the thymus. For example, one recent study demonstrated a way to visualize nerves in peripheral immune tissues (based on iDISCO clearing methods), including the thymus (81). This study reported vast TH^+^ sympathetic innervation and scarcity of VChAT^+^ parasympathetic nerves; however, no in-depth characterization of innervation was performed. Another study used an ethyl-cinnamate-based method to visualize the vascular architecture of the thymus (92). Incorporating these techniques alongside standard microscopy techniques will help provide a whole view of the thymus, identify which nerves are present, and determine the extent of thymic innervation in normal and disease states.

### Neurotransmitter quantification

Direct quantification of neurotransmitter levels in tissues can be done by a few different means. Larger neuropeptides, such as CGRP and neuropeptide Y, can be readily quantified by ELISA as these are relatively stable proteins. However, catecholamines are difficult to measure by ELISA. The preferred method for many biogenic amines, including catecholamines, is high-performance liquid chromatography (HPLC) or liquid chromatography-mass spectrometry (LCMS) (93–95). Catecholamines are also highly sensitive to rapid degradation due to high levels of monoamine oxidases in cells/tissues. Thus, samples should be isolated rapidly and flash-frozen in liquid nitrogen. One challenge with these neurotransmitter quantification techniques is that they typically require whole thymus homogenization, and as such, they do not provide any spatial information. The interpretation of the results may thus be confounded by neurotransmitter molecules stored inside cells or nerves, in addition to those released into the extracellular compartment.

## Outstanding questions

**Figure 3:**
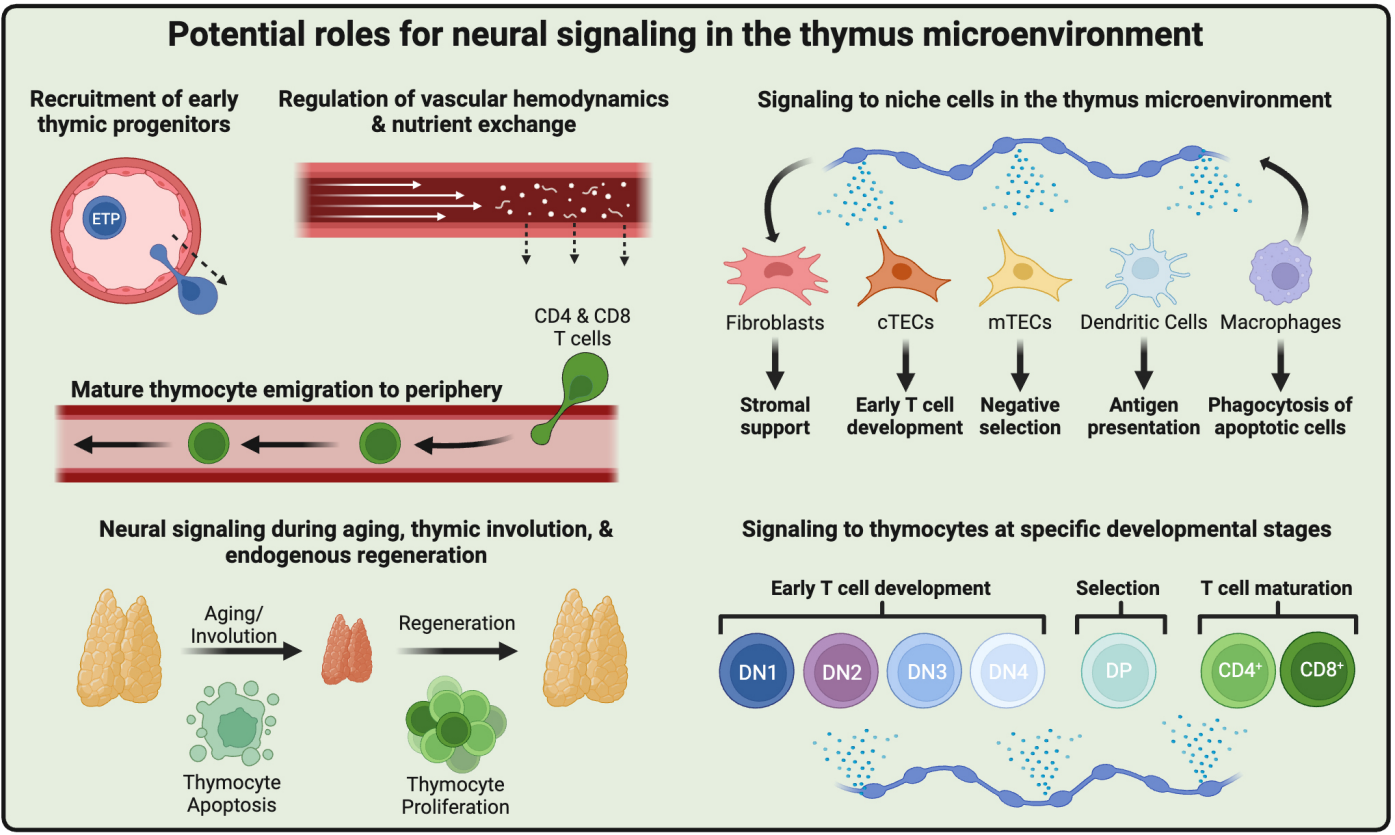
Roles for neural signaling in the thymic microenvironment. Close association of sympathetic nerves with thymic vasculature suggests potential roles in cell migration, such as ETP recruitment and mature thymocyte egress. Neural signals may also regulate blood flow and nutrient exchange at the blood-thymus barrier. Neurotransmitters may signal to thymic niche cell types, including fibroblasts, cTECs, mTECs, dendritic cells, and macrophages, and alter their associated functions. Further, nerves may signal directly to thymocytes at specific stages of development. Neural signals may participate in thymic involution during aging or chemotherapy treatment and contribute to endogenous regeneration. Created with BioRender.com.

The importance of neural signaling to immune function has become clear over the past 20-30 years (96). Hematopoietic and lymphoid tissues, such as the spleen, bone marrow, and lymph nodes, are under the direct control of the nervous system. The majority of studies have focused on mature immune cell function in specific disease states, whereas less is known about neural regulation of T cell development in the thymus. While extensive advances have been made in characterizing the thymic microenvironment through single-cell technologies, this approach typically lacks data from nerves and their signals. Below, we summarize key research areas that could help move the field forward (**Figure 3**).

### In-depth characterization of neural innervation in thymic compartments

The thymus microenvironment is highly organized, with distinct cell types and regions responsible for specific aspects of T cell development (see above). Early characterization experiments did a remarkable job demonstrating the distribution of sympathetic innervation in the thymus, including its contextual association with large blood vessels, the thymic capsule, and the subcapsular cortex. However, additional characterization would help uncover more in-depth structural and functional relationships with the heterogenous intrathymic components. One controversy is the precise trajectories and the depths to which SNS, and possibly sensory or PNS, innervation, and signaling networks extend within the thymus. It is also unclear if the nerves of the thymus that project into the subcortical parenchyma are the same as those that innervate the large blood vessels. Combined with genetic reporter models for more accurate nerve representation, new methods for whole tissue immunolabeling and imaging (discussed above) will provide definitive evidence of nerve-thymic stromal cell interactions.

### Neural regulation of thymic vasculature

The vascular network within the thymus is intricate, dense, and diverse, composed of large arteries and veins, medium arterioles and venules, smaller capillaries, and auxiliary and anastomosing vasculature systems (97). Although innervation has been generally observed in larger vessels, the precise nature and functions of these vessels are not well-established, nor how they maintain their innervation. The close association of sympathetic nerves with vessels and their biased/preferential extension toward the cortical zones suggests a potential role in regulating blood flow, the thymus-blood barrier, nutrient exchange, and cellular trafficking into and out of the thymus. Neural signals may control influx of ETP homing and efflux of mature thymocytes to secondary lymphoid organs, two processes tightly controlled by blood vessels and their unique perivascular niches in the thymus (98, 99). The SNS has an established role in mobilizing hematopoietic progenitors from the bone marrow (69, 72, 73, 100), the recruitment of leukocytes to peripheral tissues (101), and the function of mature lymphocytes in lymph nodes (82, 102, 103). Thus, SNS innervation of the thymic vasculature may serve similar roles.

### Neural regulation of the thymic microenvironment

The thymus contains a growing diversity of cell types within the thymic microenvironment. cTECs are critical for early T cell development, forming a niche where direct cell contact and paracrine signals contribute to T lineage restriction (1). T cells then undergo specific differentiation and maturation steps, including T cell receptor gene rearrangement and positive/negative selection that requires TECs and other antigen-presenting cells, including dendritic cells (6). Neural signals could help promote or inhibit some of these key roles by TECs, dendritic cells, or other thymic niche cells. For example, thymic mast cells and macrophages have been suggested to have a functional relationship with nerves due to their localization near sympathetic and peptidergic nerves (84, 104). Yet, whether these cell types respond to nerve signals is speculative, with little or no data demonstrating a functional role. Furthermore, the thymic microenvironment may also signal back to nerves, affecting innervation, axon degeneration/regeneration, or neuronal activity (43).

### Direct neural signaling to developing T cells

Many *in vitro* experiments have suggested direct neural signaling to thymocytes. As discussed, the major issue with these studies is the lack of niche signals provided by other cell types. Further, neurotransmitter receptor expression may differ depending on the stage of thymocyte differentiation. Understanding how each stage of T lineage differentiation responds to neural signals is a critical area of investigation moving forward. Using new technologies to separate specific thymocyte subsets or single-cell technologies may help generate refined and granular data. The challenging questions are how alterations in T cell development in the thymus due to changes in neural tone affect T cell function and how intrathymic neural signaling coordinates T cell development and long-term systemic immunity.

### Innervation and function during aging, thymic involution, and endogenous regeneration

Most studies of the thymus focus on preventing aging-related changes, particularly thymic involution (105), to preserve an increased TCR repertoire diversity immune system competence later in life. Recent studies have emphasized the occurrence of epithelial cell defects in thymic aging and regeneration (106). Previous studies have shown that neural innervation is maintained in the aged thymus (see above), but whether functional neural signaling remains intact is unclear. For example, some studies find altered neurotransmitter levels in the aged thymus (64, 66, 67), suggesting that neural signaling networks within the aged thymus may be altered. Furthermore, genotoxic treatments, such as chemotherapy given to cancer patients or irradiation in the context of hematopoietic stem cell transplantation, promote early thymic involution and subsequent regeneration (107). Given that SNS nerves are critical for hematopoietic regeneration in the bone marrow (70), assessing whether it functions similarly in the thymus will be essential.

## Summary

The thymus is a critical primary lymphoid organ necessary for developing T cells throughout life. Historical studies dating back to the early 20^th^ century have documented thymic neural innervation, but to date, the physiological significance of this innervation has been understudied. While the SNS is often associated with the acute stress response, this perspective oversimplifies its broader role in tissue homeostasis. Sympathetic nerves reside in all hematopoietic and lymphoid tissues, suggesting they contribute to immune development and function, even during non-stress conditions. Despite these indications, the role of neural signaling in thymic function, particularly in T cell development, remains a frontier with many unanswered questions. In this review, we summarized a large body of literature, addressing the evidence supporting neural regulation of the thymic microenvironment and the controversies and gaps that persist. Unlocking these mysteries will require harnessing advanced technologies and techniques from neuroscience and immunology, such as high-resolution whole-tissue imaging, optogenetics or chemogenetics, and single-cell transcriptomics. Progress in this field hinges on interdisciplinary collaboration, sharing of resources, and cultivating expertise that bridges disciplines. These efforts will be essential to unravel the complex interplay between the nervous system and thymic function, deepening our understanding of neuroimmune regulation in health and disease.

## References

[1] Kadouri N Nevo S Goldfarb Y Abramson J. Thymic epithelial cell heterogeneity: TEC by TEC. Nat Rev Immunol. (2019) 20:239–53. doi: 10.1038/s41577-019-0238-0 31804611

[2] James KD Cosway EJ Parnell SM White AJ Jenkinson WE Anderson G. Assembling the thymus medulla: Development and function of epithelial cell heterogeneity. BioEssays. (2024) 46:2300165. doi: 10.1002/bies.202300165 38161233 PMC11475500

[3] Nusser A Sagar Swann JB Krauth B Diekhoff D Calderon L. Developmental dynamics of two bipotent thymic epithelial progenitor types. Nature. (2022) 606:165–71. doi: 10.1038/s41586-022-04752-8 PMC9159946 35614226

[4] Michelson DA Hase K Kaisho T Benoist C Mathis D. Thymic epithelial cells co-opt lineage-defining transcription factors to eliminate autoreactive T cells. Cell. (2022) 185:2542–2558.e18. doi: 10.1016/j.cell.2022.05.018 35714609 PMC9469465

[5] Castañeda J Hidalgo Y Sauma D Rosemblatt M Bono MR Núñez S. The multifaceted roles of B cells in the thymus: from immune tolerance to autoimmunity. Front Immunol. (2021) 12:766698. doi: 10.3389/fimmu.2021.766698 34790201 PMC8591215

[6] Breed ER Vobořil M Ashby KM Martinez RJ Qian L Wang H. Type 2 cytokines in the thymus activate Sirpα+ dendritic cells to promote clonal deletion. Nat Immunol. (2022) 23:1042–51. doi: 10.1038/s41590-022-01218-x PMC10037932 35637352

[7] Nitta T. Mesenchymal stromal cells in the thymus. Inflammation Regener. (2022) 42:1–8. doi: 10.1186/s41232-022-00219-5 PMC9628078 36320070

[8] Nitta T Ota A Iguchi T Muro R Takayanagi H. The fibroblast: An emerging key player in thymic T cell selection. Immunol Rev. (2021) 302:68–85. doi: 10.1111/imr.12985 34096078 PMC8362222

[9] Nitta T Takayanagi H. Non-epithelial thymic stromal cells: unsung heroes in thymus organogenesis and T cell development. Front Immunol. (2021) 11:620894. doi: 10.3389/fimmu.2020.620894 33519827 PMC7840694

[10] Nevo S Frenkel N Kadouri N Gome T Rosenthal N Givony T. Tuft cells and fibroblasts promote thymus regeneration through ILC2-mediated type 2 immune response. Sci Immunol. (2024) 9(91). doi: 10.1126/sciimmunol.abq6930 38215193

[11] Pines DL Majman R. The innervation of the thymus. J Nerv Ment Dis. (1929) 69:361–84. https://journals.lww.com/jonmd/citation/1929/04000/the_innervation_of_the_thymus.1.aspx (Accessed November 20, 2024).

[12] Williams JM Felten DL. Sympathetic innervation of murine thymus and spleen: A comparative histofluorescence study. Anat Rec. (1981) 199:531–42. doi: 10.1002/ar.1091990409 6168212

[13] Bulloch K Pomerantz W. Autonomic nervous system innervation of thymic-related lymphoid tissue in wildtype and nude mice. J Comp Neurol. (1984) 228:57–68. doi: 10.1002/cne.902280107 6480908

[14] Felten D Felten S Carlson S Olschowka J Livnat S. Noradrenergic and peptidergic innervation of lymphoid tissue. J Immunol. (1985) 135:755–65. doi: 10.4049/jimmunol.135.2.755 2861231

[15] Bulloch K Moore RY. Innervation of the thymus gland by brain stem and spinal cord in mouse and rat. Am J Anat. (1981) 162:157–66. doi: 10.1002/aja.1001620207 7304470

[16] Nance DM Hopkins DA Bieger D. Re-investigation of the innervation of the thymus gland in mice and rats. Brain Behav Immun. (1987) 1:134–47. doi: 10.1016/0889-1591(87)90016-X 2454693

[17] Tollefson L Bulloch K. Dual-label retrograde transport: CNS innervation of the mouse thymus distinct from other mediastinum viscera. J Neurosci Res. (1990) 25:20–8. doi: 10.1002/jnr.490250104 2319621

[18] Trotter RN Stornetta RL Guyenet PG Roberts MR. Transneuronal mapping of the CNS network controlling sympathetic outflow to the rat thymus. Auton Neurosci. (2007) 131:9–20. doi: 10.1016/j.autneu.2006.06.001 16843070

[19] Kenney MJ Weiss ML Haywood JR. The paraventricular nucleus: An important component of the central neurocircuitry regulating sympathetic nerve outflow. Acta Physiol Scand. (2003) 177:7–15. doi: 10.1046/j.1365-201X.2003.01042.x 12492774

[20] Kendall MD Al-Shawaf AA. Innervation of the rat thymus gland. Brain Behav Immun. (1991) 5:9–28. doi: 10.1016/0889-1591(91)90004-T 1648986

[21] Kurz B von Gaudecker B Kranz A Krisch B Mentlein R. Calcitonin gene-related peptide and its receptor in the thymus. Peptides (NY). (1995) 16:1497–503. doi: 10.1016/0196-9781(95)02042-X 8745064

[22] Kranz A Kendall MD Von Gaudecker B. Studies on rat and human thymus to demonstrate immunoreactivity of calcitonin gene-related peptide, tyrosine hydroxylase and neuropeptide Y. J Anat. (1997) 191:441–50. doi: 10.1046/j.1469-7580.1997.19130441.x PMC1467701 9419001

[23] Jacobson A Yang D Vella M Chiu IM. The intestinal neuro-immune axis: crosstalk between neurons, immune cells, and microbes. Mucosal Immunol. (2021) 14:555–65. doi: 10.1038/s41385-020-00368-1 PMC8075967 33542493

[24] Yang D Almanzar N Chiu IM. The role of cellular and molecular neuroimmune crosstalk in gut immunity. Cell Mol Immunol. (2023) 20:1259–69. doi: 10.1038/s41423-023-01054-5 PMC10616093 37336989

[25] Al-Shalan HAM Hu D Nicholls PK Greene WK Ma B. Immunofluorescent characterization of innervation and nerve-immune cell neighborhood in mouse thymus. Cell Tissue Res. (2019) 378:239–54. doi: 10.1007/s00441-019-03052-4 31230166

[26] Zhang B Vogelzang A Miyajima M Sugiura Y Wu Y Chamoto K. B cell-derived GABA elicits IL-10+ macrophages to limit anti-tumour immunity. Nature. (2021) 599:471–6. doi: 10.1038/s41586-021-04082-1 PMC8599023 34732892

[27] Schloss MJ Hulsmans M Rohde D Lee IH Severe N Foy BH. B lymphocyte-derived acetylcholine limits steady-state and emergency hematopoiesis. Nat Immunol. (2022) 23:605–18. doi: 10.1038/s41590-022-01165-7 PMC8989652 35352063

[28] Shao L Elujoba-Bridenstine A Zink KE Sanchez LM Cox BJ Pollok KE. The neurotransmitter receptor Gabbr1 regulates proliferation and function of hematopoietic stem and progenitor cells. Blood. (2021) 137:775–87. doi: 10.1182/blood.2019004415 PMC7885825 32881992

[29] Rinner I Globerson A Kawashima K Korsatko W Schauenstein K. A possible role for acetylcholine in the dialogue between thymocytes and thymic stroma. Neuroimmunomodulation. (1999) 6:51–5. doi: 10.1159/000026364 9876235

[30] Francelin C Veneziani LP Farias A dos S Mendes-da-Cruz DA Savino W. Neurotransmitters modulate intrathymic T-cell development. Front Cell Dev Biol. (2021) 9:668067. doi: 10.3389/fcell.2021.668067 33928093 PMC8076891

[31] Panneck AR Rafiq A Schütz B Soultanova A Deckmann K Chubanov V. Cholinergic epithelial cell with chemosensory traits in murine thymic medulla. Cell Tissue Res. (2014) 358:737–48. doi: 10.1007/s00441-014-2002-x PMC4233111 25300645

[32] Givony T Leshkowitz D Del Castillo D Nevo S Kadouri N Dassa B. Thymic mimetic cells function beyond self-tolerance. Nature. (2023) 622:1–9. doi: 10.1038/s41586-023-06512-8 37674082

[33] Baran-Gale J Morgan MD Maio S Dhalla F Calvo-Asensio I Deadman ME. Ageing compromises mouse thymus function and remodels epithelial cell differentiation. Elife. (2020) 9:1–71. doi: 10.7554/ELIFE.56221 PMC7490013 32840480

[34] Warthan MD Freeman JG Loesser KE Lewis CW Hong M Conway CM. Phenylethanolamine N-methyl transferase expression in mouse thymus and spleen. Brain Behav Immun. (2002) 16:493–9. doi: 10.1006/brbi.2001.0637 12096893

[35] Xing L Guo J Wang X. Induction and expression of β-calcitonin gene-related peptide in rat T lymphocytes and its significance. J Immunol. (2000) 165:4359–66. doi: 10.4049/JIMMUNOL.165.8.4359 11035072

[36] Lifantseva NV Koneeva TO Voronezhskaya EE Melnikova VI. Expression of components of the serotonergic system in the developing rat thymus. Dokl Biochem Biophys. (2017) 477:401–4. doi: 10.1134/S1607672917060151 29297119

[37] Lifantseva NV Koneeva TO Voronova SN Zakharova LA Melnikova VI. The inhibition of dopamine synthesis in fetuses changes the pattern of T-lymphocyte maturation in the thymus of adult rats. Dokl Biochem Biophys. (2016) 470:342–4. doi: 10.1134/S1607672916050082 27817026

[38] Stefulj J Jernej B Cicin-Sain L Rinner I Schauenstein K. mRNA expression of serotonin receptors in cells of the immune tissues of the rat. Brain Behav Immun. (2000) 14:219–24. doi: 10.1006/brbi.1999.0579 10970681

[39] Storto M De Grazia U Battaglia G Felli MP Maroder M Gulino A. Expression of metabotropic glutamate receptors in murine thymocytes and thymic stromal cells. J Neuroimmunol. (2000) 109:112–20. doi: 10.1016/S0165-5728(00)00269-1 10996213

[40] Pešić V Kosec D Radojević K Pilipović I Perišić M Vidić-Danković B. Expression of α1-adrenoceptors on thymic cells and their role in fine tuning of thymopoiesis. J Neuroimmunol. (2009) 214:55–66. doi: 10.1016/j.jneuroim.2009.06.018 19646768

[41] Pešić V Plećaš-Solarović B Radojević K Kosec D Pilipović I Perišić M. Long-term β-adrenergic receptor blockade increases levels of the most mature thymocyte subsets in aged rats. Int Immunopharmacol. (2007) 7:674–86. doi: 10.1016/j.intimp.2007.01.017 17386415

[42] Vizi ES Orsó E Osipenko ON Haskó G Elenkov IJ. Neurochemical, electrophysiological and immunocytochemical evidence for a noradrenergic link between the sympathetic nervous system and thymocytes. Neuroscience. (1995) 68:1263–76. doi: 10.1016/0306-4522(95)00215-5 8544999

[43] Haskó G Elenkov IJ Vizi ES. Presynaptic receptors involved in the modulation of release of noradrenaline from the sympathetic nerve terminals of the rat thymus. Immunol Lett. (1995) 47:133–7. doi: 10.1016/0165-2478(95)00085-J 8537090

[44] Brewer JA Kanagawa O Sleckman BP Muglia LJ. Thymocyte apoptosis induced by T cell activation is mediated by glucocorticoids *in vivo* . J Immunol. (2002) 169:1837–43. doi: 10.4049/jimmunol.169.4.1837 12165507

[45] Gu C Ma YC Benjamin J Littman D Chao MV Huang XY. Apoptotic signaling through the α-adrenergic receptor: A new G(s) effector pathway. J Biol Chem. (2000) 275:20726–33. doi: 10.1074/jbc.M000152200 10767282

[46] Wajeman-Chao SA Lancaster SA Graf LH Chambers DA. Mechanism of catecholamine-mediated destabilization of messenger RNA encoding thy-1 protein in T-lineage cells. J Immunol. (1998) 161:4825–33. doi: 10.4049/jimmunol.161.9.4825 9794415

[47] LaJevic MD Koduvayur SP Caffrey V Cohen RL Chambers DA. Thy-1 mRNA destabilization by norepinephrine a 3’ UTR cAMP responsive decay element and involves RNA binding proteins. Brain Behav Immun. (2010) 24:1078–88. doi: 10.1016/j.bbi.2010.04.006 PMC2939224 20412850

[48] Cook-Mills JM Cohen RL Perlman RL Chambers DA. Inhibition of lymphocyte activation by catecholamines: evidence for a non-classical mechanism of catecholamine action. Immunology. (1995) 85:544–9. PMC1383781 7558147

[49] Medina S Del Río M Hernanz A de la Fuente M. The NPY effects on murine leukocyte adherence and chemotaxis change with age: Adherent cell implication. Regul Pept. (2000) 95:35–45. doi: 10.1016/S0167-0115(00)00134-8 11062330

[50] Von Patay B Kurz B Mentlein R. Effect of transmitters and co-transmitters of the sympathetic nervous system on interleukin-6 synthesis in thymic epithelial cells. Neuroimmunomodulation. (1999) 6:45–50. doi: 10.1159/000026363 9876234

[51] Galbiati F Basso V Cantuti L Givogri MI Lopez-Rosas A Perez N. Autonomic denervation of lymphoid organs leads to epigenetic immune atrophy in a mouse model of Krabbe disease. J Neurosci. (2007) 27:13730–8. doi: 10.1523/JNEUROSCI.3379-07.2007 PMC6673629 18077684

[52] Tsunokuma N Tetteh DN Isono K Kuniishi-Hikosaka M Tsuneto M Ishii K. Depletion of neural crest–derived cells leads to plasma noradrenaline decrease and alters T cell development. J Immunol. (2023) 211:1494–505. doi: 10.4049/jimmunol.2300045 37747298

[53] Tsao CW Cheng JT Shen CL Lin YS. 6-Hydroxydopamine induces thymocyte apoptosis in mice. J Neuroimmunol. (1996) 65:91–5. doi: 10.1016/0165-5728(95)00166-2 8964900

[54] Hall NR McClure JE Hu SK Tare NS Seals CM Goldstein AL. Effects of 6-hydroxydopamine upon primary and secondary thymus dependent immune responses. Immunopharmacology. (1982) 5:39–48. doi: 10.1016/0162-3109(82)90035-2 6813289

[55] Solti I Kvell K Talaber G Veto S Acs P Gallyas F. Thymic atrophy and apoptosis of CD4+CD8+ Thymocytes in the cuprizone model of multiple sclerosis. PloS One. (2015) 10:e0129217. doi: 10.1371/JOURNAL.PONE.0129217 26053248 PMC4460035

[56] Martin NA Molnar V Szilagyi GT Elkjaer ML Nawrocki A Okarmus J. Experimental demyelination and axonal loss are reduced in MicroRNA-146a deficient mice. Front Immunol. (2018) 9:490/BIBTEX. doi: 10.3389/FIMMU.2018.00490/BIBTEX 29593734 PMC5857529

[57] Das Neves SP Serre-Miranda C Nobrega C Roque S Cerqueira JJ Correia-Neves M. Immune thymic profile of the MOG-induced experimental autoimmune encephalomyelitis mouse model. Front Immunol. (2018) 9:2335/BIBTEX. doi: 10.3389/FIMMU.2018.02335/BIBTEX 30369926 PMC6194318

[58] Almuslehi MSM Sen MK Shortland PJ Mahns DA Coorssen JR. CD8 T-cell recruitment into the central nervous system of cuprizone-fed mice: relevance to modeling the etiology of multiple sclerosis. Front Cell Neurosci. (2020) 14:43/BIBTEX. doi: 10.3389/FNCEL.2020.00043/BIBTEX 32210765 PMC7076139

[59] Jiang Q Ma X Zhu G Si W He L Yang G. Altered T cell development in an animal model of multiple sclerosis. Exp Neurol. (2024) 371:114579. doi: 10.1016/J.EXPNEUROL.2023.114579 37866699

[60] Francelin C Borin A Funari J Pradella F Santos LMB Savino W. Thymic innervation impairment in experimental autoimmune encephalomyelitis. Neuroimmunomodulation. (2023) 31:25–39. doi: 10.1159/000535859 38128499

[61] Maryanovich M Zahalka AH Pierce H Pinho S Nakahara F Asada N. Adrenergic nerve degeneration in bone marrow drives aging of the hematopoietic stem cell niche. Nat Med. (2018) 24:782–91. doi: 10.1038/s41591-018-0030-x PMC6095812 29736022

[62] Ho Y-H del Toro R Rivera-Torres J Rak J Korn C García-García A. Remodeling of bone marrow hematopoietic stem cell niches promotes myeloid cell expansion during premature or physiological aging. Cell Stem Cell. (2019) 25(3):407–18. doi: 10.1016/j.stem.2019.06.007 PMC6739444 31303548

[63] Carpenter RS Maryanovich M. Systemic and local regulation of hematopoietic homeostasis in health and disease. Nat Cardiovasc Res. (2024) 3:1–15. doi: 10.1038/s44161-024-00482-4 39196230

[64] Madden KS Bellinger DL Felten SY Snyder E Maida ME Felten DL. Alterations in sympathetic innervation of thymus and spleen in aged mice. Mech Ageing Dev. (1997) 94:165–75. doi: 10.1016/S0047-6374(96)01858-1 9147368

[65] Bellinger DL Felten SY Felten DL. Maintenance of noradrenergic sympathetic innervation in the involuted thymus of the aged Fischer 344 rat. Brain Behav Immun. (1988) 2:133–50. doi: 10.1016/0889-1591(88)90014-1 3233357

[66] ThyagaRajan S Madden KS Teruya B Stevens SY Felten DL Bellinger DL. Age-associated alterations in sympathetic noradrenergic innervation of primary and secondary lymphoid organs in female Fischer 344 rats. J Neuroimmunol. (2011) 233:54–64. doi: 10.1016/j.jneuroim.2010.11.012 21186063 PMC3074019

[67] Cavallotti C Artico M Cavallotti D. Occurrence of adrenergic nerve fibers and of noradrenaline in thymus gland of juvenile and aged rats. Immunol Lett. (1999) 70:53–62. doi: 10.1016/S0165-2478(99)00127-3 10541052

[68] Madden KS Felten DL. Beta-adrenoceptor blockade alters thymocyte differentiation in aged mice. Cell Mol Biol. (2001) 47:189–96. 11292254

[69] Méndez-Lucas D Battista M Frenette PS. Haematopoietic stem cell release is regulated by circadian oscillations. Nature. (2008) 452:442–7. doi: 10.1038/nature06685 18256599

[70] Lucas D Scheiermann C Chow A Kunisaki Y Bruns I Barrick C. Chemotherapy-induced bone marrow nerve injury impairs hematopoietic regeneration. Nat Med. (2013) 19:695–703. doi: 10.1038/nm.3155 23644514 PMC3964478

[71] Nevin JT Moussa M Corwin WL Mandoiu II Srivastava PK. Sympathetic nervous tone limits the development of myeloid-derived suppressor cells. Sci Immunol. (2020) 5:9368. doi: 10.1126/SCIIMMUNOL.AAY9368 32917793

[72] Katayama Y Battista M Kao WM Hidalgo A Peired AJ Thomas SA. Signals from the sympathetic nervous system regulate hematopoietic stem cell egress from bone marrow. Cell. (2006) 124:407–21. doi: 10.1016/j.cell.2005.10.041 16439213

[73] Dar A Schajnovitz A Lapid K Kalinkovich A Itkin T Ludin A. Rapid mobilization of hematopoietic progenitors by AMD3100 and catecholamines is mediated by CXCR4-dependent SDF-1 release from bone marrow stromal cells. Leukemia. (2011) 25:1286–96. doi: 10.1038/leu.2011.62 PMC4175714 21494253

[74] Kárai LJ Russell JT Iadarola MJ Oláh Z. Vanilloid receptor 1 regulates multiple calcium compartments and contributes to ca2+-induced ca2+ Release in sensory neurons. J Biol Chem. (2004) 279:16377–87. doi: 10.1074/jbc.M310891200 14963041

[75] Raisinghani M Pabbidi RM Premkumar LS. Activation of transient receptor potential vanilloid 1 (TRPV1) by resiniferatoxin. J Physiol. (2005) 567:771–86. doi: 10.1113/jphysiol.2005.087874 PMC1474234 16037081

[76] Gao X Zhang D Xu C Li H Caron KM Frenette PS. Nociceptive nerves regulate haematopoietic stem cell mobilization. Nature. (2021) 589:591–6. doi: 10.1038/s41586-020-03057-y PMC7856173 33361809

[77] Hou Y Sun L LaFleur MW Huang L Lambden C Thakore PI. Neuropeptide signalling orchestrates T cell differentiation. Nature. (2024) 635:1–9. doi: 10.1038/s41586-024-08049-w PMC11951087 39415015

[78] Wachter SB Gilbert EM. Beta-adrenergic receptors, from their discovery and characterization through their manipulation to beneficial clinical application. Cardiol (Switzerland). (2012) 122:104–12. doi: 10.1159/000339271 22759389

[79] Scarlett-Ferguson H. Adrenergic drugs. In: Clinical drug Therapy for Canadian Practice, 2nd ed. Treasure Island, Florida, USA: StatPearls Publishing (2011). p. 277–91. doi: 10.5005/jp/books/14244_9

[80] Saito M Iwawaki T Taya C Yonekawa H Noda M Inui Y. Diphtheria toxin receptor-mediated conditional and targeted cell ablation in transgenic mice. Nat Biotechnol. (2001) 19:746–50. doi: 10.1038/90795 11479567

[81] Ding X Wang H Qian X Han X Yang L Cao Y. Panicle-shaped sympathetic architecture in the spleen parenchyma modulates antibacterial innate immunity. Cell Rep. (2019) 27:3799–3807.e3. doi: 10.1016/j.celrep.2019.05.082 31242414

[82] Chen CS Weber J Holtkamp SJ Ince LM de Juan A Wang C. Loss of direct adrenergic innervation after peripheral nerve injury causes lymph node expansion through IFN-γ. J Exp Med. (2021) 218(8). doi: 10.1084/jem.20202377 PMC8185988 34086056

[83] Elkhatib SK Moshfegh CM Watson GF Schwab AD Katsurada K Patel KP. Splenic denervation attenuates repeated social defeat stress-induced T lymphocyte inflammation. Biol Psychiatry Global Open Sci. (2021) 1:190–200. doi: 10.1016/j.bpsgos.2021.05.004 PMC8941638 35330608

[84] Artico M Cavallotti C Cavallotti D. Adrenergic nerve fibres and mast cells: Correlation in rat thymus. Immunol Lett. (2002) 84:69–76. doi: 10.1016/S0165-2478(02)00145-1 12161286

[85] Montgomery KL Iyer SM Christensen AJ Deisseroth K Delp SL. Beyond the brain: Optogenetic control in the spinal cord and peripheral nervous system. Sci Transl Med. (2016) 8(337). doi: 10.1126/scitranslmed.aad7577 27147590

[86] Kim CK Adhikari A Deisseroth K. Integration of optogenetics with complementary methodologies in systems neuroscience. Nat Rev Neurosci. (2017) 18:222–35. doi: 10.1038/nrn.2017.15 PMC5708544 28303019

[87] Roth BL. DREADDs for neuroscientists. Neuron. (2016) 89:683–94. doi: 10.1016/j.neuron.2016.01.040 PMC4759656 26889809

[88] Zhang S Gumpper RH Huang XP Liu Y Krumm BE Cao C. Molecular basis for selective activation of DREADD-based chemogenetics. Nature. (2022) 612:354–62. doi: 10.1038/s41586-022-05489-0 36450989

[89] Saleeba C Dempsey B Le S Goodchild A McMullan S. A student’s guide to neural circuit tracing. Front Neurosci. (2019) 13:897. doi: 10.3389/fnins.2019.00897 31507369 PMC6718611

[90] Ueda HR Ertürk A Chung K Gradinaru V Chédotal A Tomancak P. Tissue clearing and its applications in neuroscience. Nat Rev Neurosci. (2020) 21:61–79. doi: 10.1038/s41583-019-0250-1 31896771 PMC8121164

[91] Weiss KR Voigt FF Shepherd DP Huisken J. Tutorial: practical considerations for tissue clearing and imaging. Nat Protoc. (2021) 16:2732–48. doi: 10.1038/s41596-021-00502-8 PMC10542857 34021294

[92] Sun Q Tiziana P Khan A ul M Heuveline V Gretz N. A simple optical tissue clearing pipeline for 3D vasculature imaging of the mediastinal organs in mice. Int J Exp Pathol. (2021) 102:218–27. doi: 10.1111/iep.12399 PMC8576639 34613652

[93] Holm TH Isaksen TJ Lykke-Hartmann K. HPLC neurotransmitter analysis. Methods Mol Biol. (2016) 1377:333–40. doi: 10.1007/978-1-4939-3179-8_29 26695044

[94] Kim TH Choi J Kim HG Kim HR. Quantification of neurotransmitters in mouse brain tissue by using liquid chromatography coupled electrospray tandem mass spectrometry. J Anal Methods Chem. (2014) 2014:506870. doi: 10.1155/2014/506870 25258696 PMC4166658

[95] Guiard BP Gotti G. The high-precision liquid chromatography with electrochemical detection (HPLC-ECD) for monoamines neurotransmitters and their metabolites: A review. Molecules. (2024) 29:496. doi: 10.3390/molecules29020496 38276574 PMC10818480

[96] Schiller M Ben-Shaanan TL Rolls A. Neuronal regulation of immunity: why, how and where? Nat Rev Immunol. (2021) 21:20–36. doi: 10.1038/s41577-020-0387-1 32811994

[97] Irino S Takasugi N Murakami T. Vascular architecture of thymus and lymph nodes: Blood vessels, transmural passage of lymphocytes, and cell-interactions. Scan Electron Microsc. (1981) 1981:89–98. 7330596

[98] Zlotoff DA Bhandoola A. Hematopoietic progenitor migration to the adult thymus. Ann N Y Acad Sci. (2011) 1217:122–38. doi: 10.1111/J.1749-6632.2010.05881.X PMC3076003 21251013

[99] Ruiz Pérez M Vandenabeele P Tougaard P. The thymus road to a T cell: migration, selection, and atrophy. Front Immunol. (2024) 15:1443910/BIBTEX. doi: 10.3389/FIMMU.2024.1443910/BIBTEX 39257583 PMC11384998

[100] Méndez-Battista M Frenette PS. Cooperation of β2- and β3-adrenergic receptors in hematopoietic progenitor cell mobilization. Ann N Y Acad Sci. (2010) 1192:139–44. doi: 10.1111/j.1749-6632.2010.05390.x PMC4106131 20392229

[101] Scheiermann C Kunisaki Y Lucas D Chow A Jang JE Zhang D. Adrenergic nerves govern circadian leukocyte recruitment to tissues. Immunity. (2012) 37:290–301. doi: 10.1016/j.immuni.2012.05.021 22863835 PMC3428436

[102] Druzd D Matveeva O Ince L Harrison U He W Schmal C. Lymphocyte circadian clocks control lymph node trafficking and adaptive immune responses. Immunity. (2017) 46:120–32. doi: 10.1016/j.immuni.2016.12.011 PMC5263259 28087238

[103] De Virgiliis F Oliva VM Kizil B Scheiermann C. Control of lymph node activity by direct local innervation. Trends Neurosci. (2022) 45:704–12. doi: 10.1016/j.tins.2022.06.006 35820971

[104] Müller S Weihe E. Interrelation of peptidergic innervation with mast cells and ED1-positive cells in rat thymus. Brain Behav Immun. (1991) 5:55–72. doi: 10.1016/0889-1591(91)90007-W 1648985

[105] Boehm T Swann JB. Thymus involution and regeneration: Two sides of the same coin? Nat Rev Immunol. (2013) 13:831–8. doi: 10.1038/nri3534 24052146

[106] Kousa AI Jahn L Zhao K Flores AE Acenas D Lederer E. Age-related epithelial defects limit thymic function and regeneration. Nat Immunol. (2024) 25:1593–606. doi: 10.1038/s41590-024-01915-9 PMC11362016 39112630

[107] Kinsella S Dudakov JA. When the damage is done: injury and repair in thymus function. Front Immunol. (2020) 11:1745/BIBTEX. doi: 10.3389/FIMMU.2020.01745/BIBTEX 32903477 PMC7435010

